# A Man With Intractable Back Pain, Spondylodiscitis: A Case Report of Uncommon Site of Spinal Gout

**DOI:** 10.7759/cureus.16480

**Published:** 2021-07-19

**Authors:** Imad Karam, Anam Ahmad, Donica L Baker

**Affiliations:** 1 Internal Medicine, St. Luke's Hospital, Chesterfield, USA; 2 Rheumatology, St. Luke's Hospital, Chesterfield, USA

**Keywords:** lower back pain (lbp), gout flare, spondylodiscitis, spinal gout, colchicine, allupurinol, uric acid

## Abstract

Gout typically affects the peripheral joints but uncommonly can involve the axial skeleton and rarely the intervertebral discs. We present a rare case of gouty spondylodiscitis affecting the intervertebral disc in the lumbar spine. Our patient with a gout history not on any maintenance therapy presented intractable right-sided back pain radiating to the right lower extremity. Computed tomography scan findings were consistent with spondylosis, while magnetic resonance imaging showed concern of infectious discitis. Initially, he was treated for infectious discitis with IV antibiotics. Biopsy of the L5-S1 disc space revealed monosodium urate crystals, confirming the diagnosis of gouty spondylodiscitis. He was managed with IV dexamethasone and recovered well on a tapering dose of steroids and colchicine followed by allopurinol once acute flare resolved.

## Introduction

Gout is a common disabling inflammatory arthritis characterized by intense swelling, erythema, warmth, and pain in a peripheral joint or bursa. This disease is caused by monosodium urate (MSU) nucleation and growth in the synovium of joints secondary to long-standing elevated serum urate above saturation threshold. Spinal gout was initially thought to be a rare entity, but its prevalence is increasing. The first case was reported in 1953 by Koskoff et al. [1]. This was followed by multiple cases ranging from asymptomatic to severe complications like paraplegia [2]. Spinal gout can affect any spinal structure, including facet joints, laminae, vertebral bodies, pedicles, and the soft tissues adjacent to the spinal column. Involvement of intervertebral disc has been rarely reported in the literature. It could be a site for deposition of MSU crystals and development of gout [3].

This manuscript aims to create awareness and to increase the index of suspicion of spinal gout involving intervertebral discs as a differential diagnosis of back pain diagnosis and management of spinal gout.

## Case presentation

We present a case of a 78-year-old gentleman with multiple comorbidities, including type II diabetes mellitus, chronic kidney disease (CKD), hypertension, hyperlipidemia, coronary artery disease (CAD), chronic back pain status post microdiscectomy, status post placement of dorsal column stimulator and gout presented with intractable right-sided lower back pain.

He described severe, worsening right-sided lower back pain, 10/10 in intensity radiating to the right lower extremity, exacerbated by movement associated with numbness, tingling, and difficulty in ambulation. He denied weakness, fever, chills, urinary or fecal incontinence, and saddle anesthesia. The patient had a gout flare-up in his right foot four weeks ago, for which he usually used to take steroids but could not take them due to uncontrolled diabetes. He went to the emergency department one week before the presentation, where he was diagnosed with chronic back pain and was discharged on a course of steroids.

On presentation, the musculoskeletal examination was limited due to pain. He had exquisite tenderness of the right lower back. The neurological examination was unremarkable, with normal power in limbs and no focal deficit. All other joints examination was within normal limits. Gait was normal as well. Laboratory studies showed normal complete blood count, basic metabolic panel was significant, with a creatinine of 1.8 mg/dL and a glomerular filtration rate of 37 mL/min/1.73 m^2^. C-reactive protein was 3.4 mg/L and uric acid was 11.8 mg/dL.

**Figure 1:**
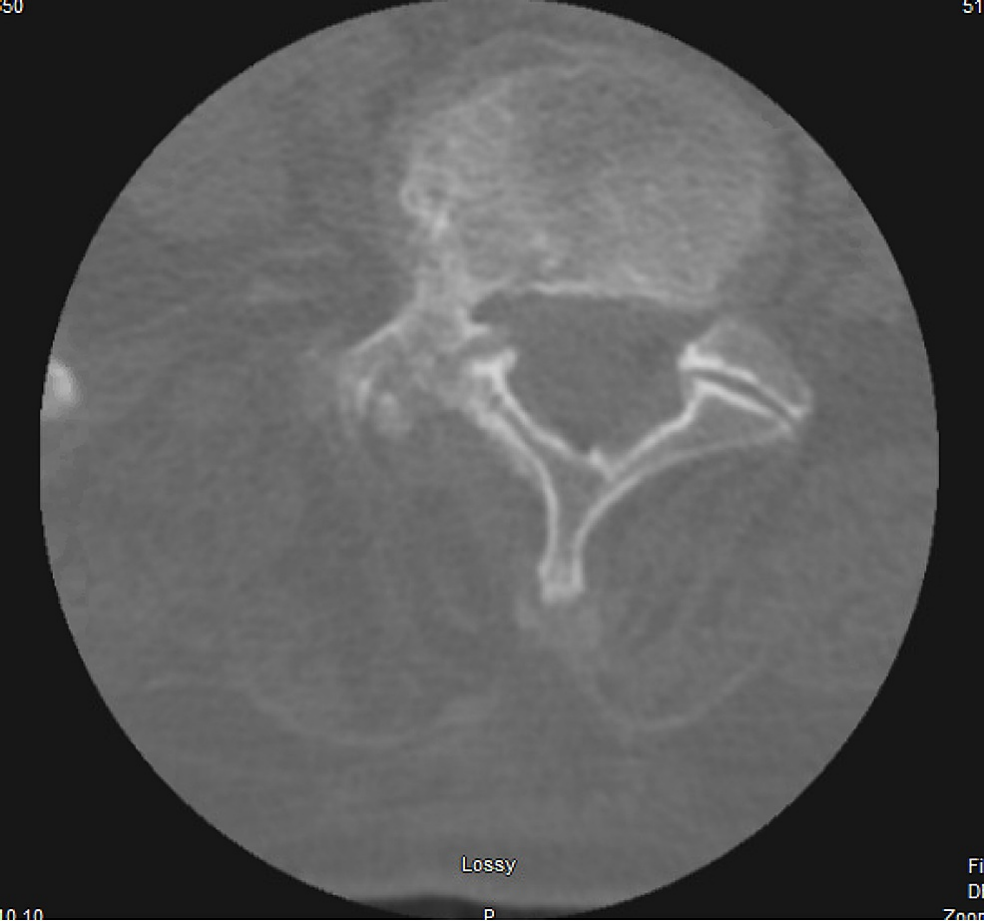
CT scan without contrast showed moderate disc bulging CT, computed tomography

**Figure 2:**
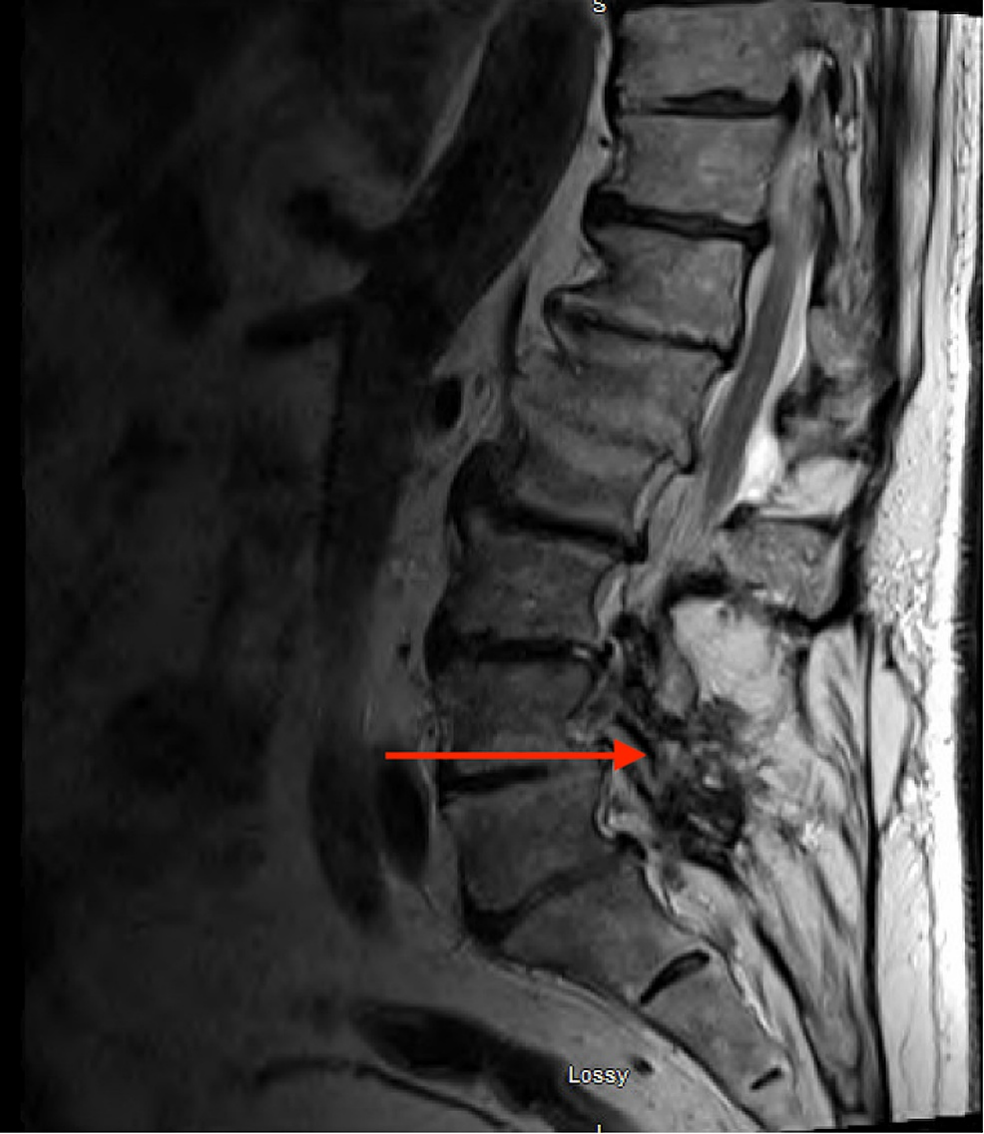
MRI spine without contrast showed disc bulging with development of fluid within the disc space and right paraspinous inflammation without the abscess MRI, magnetic resonance imaging

The pelvis's plain radiograph showed degenerative disc disease of the lumbar spine but no abnormality in the right hip. Computed tomographic (CT) scan without contrast showed moderate disc bulges at multiple levels, including L2-S1 with lateral recess stenosis and moderate-to-severe facet stenosis (Figure 1). Magnetic resonance imaging (MRI) spine showed similar results as that of CT scan but with new interval development of fluid within the disc space with endplate edema and reactive changes at level L5-S1 with right paraspinous inflammation without abscess (Figure 2). In addition, there was diffuse disc bulging, with persistent moderate left and severe right foraminal stenosis with endplate osteophyte formation. The findings were consistent with discitis without epidural or paraspinous abscess.

**Figure 3:**
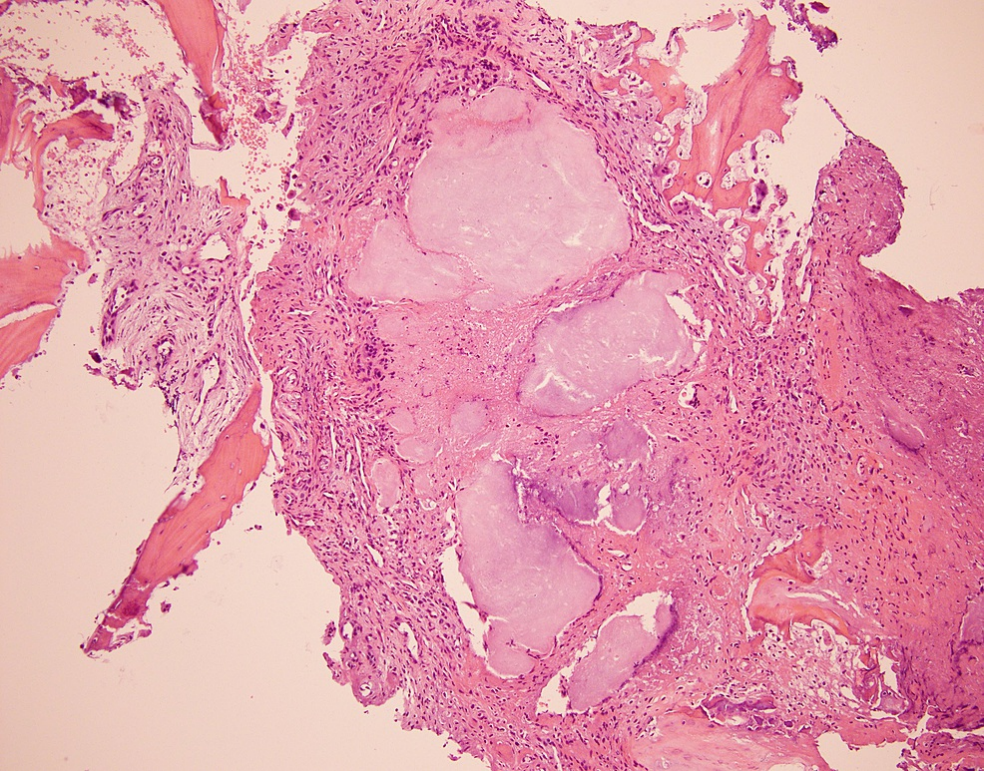
Histologic sections of L5 and S1 biopsy showed few fragments of bone and associated soft tissue, deposits of uric acid crystals (gout), accompanied by a mixed inflammatory infiltrate that includes neutrophils and histiocytes

In suspicion of infectious L5-S1 discitis, the patient was started on IV vancomycin; infectious workup was done, including urine and blood cultures, which were negative. A fluoroscopy-guided lumbar disc biopsy of the L5-S1 disc space and aspiration on microscopic examination showed deposits of uric acid crystals (gout), accompanied by a mixed inflammatory infiltrate that included neutrophils and histiocytes (Figure 3). Gram stain and cultures of the aspirate were negative.

As per biopsy results, a diagnosis of gouty spondylodiscitis was made, and he was started on IV dexamethasone 4 mg every 12 hours. The neurosurgery team consultation was sought for any possible need of surgical decompression; however, the need was obviated since the patient recovered with medical management itself. However, antibiotics were continued in anticipation of infection following gout-related necroinflammation. Upon discharge, the patient was started on an adjusted renal dose of colchicine and tapering of steroids. On follow-up appointment, he was started on allopurinol after resolution of an acute gout flare, and repeat uric acid was 5.1 mg/dL a few months later, and his back pain resolved.

## Discussion

Gout usually involves peripheral joints, but axial involvement is still an uncommon presentation. Although no studies have concluded about the exact mechanism of axial involvement, an increase in uric acid in blood has been thought to increase uric acid in cerebrospinal fluid, leading to obstruction of canal and foramen [4].

Spinal gout more commonly involves the lumbar spine, followed by the thoracic and cervical spine [5]. The most common presentation is back pain with radiculopathy and neurogenic contraction. Our patient presented with intractable radiculopathy. Although while keeping this differential diagnosis in mind, we should also rule out other conditions, including disc herniation, infection, and tumor.

Risk factors for spinal gout are the same as that of peripheral gout. New evidence suggests that the triad of diabetes, hypertension, and atherosclerosis may result in higher physiologically tolerable uric acid levels [6]. Our patient has a history of uncontrolled diabetes, hypertension, and CAD; thus, all the factors put him at high risk of developing gout.

For diagnosis, different imaging modalities exist. Conventional radiography has a sensitivity of 31%. The radiograph findings like punched-out erosions, overhanging edges, and soft-tissue calcifications are more consistent with chronic tophaceous gout. CT scan can detect deeper intraarticular and intraosseous tophi. A dual-energy CT is an advanced modality that has high sensitivity to identify tophaceous gout even with subclinical urate burden and imaging multiple joints at the same time [7]. In comparison, MRI can show subclinical inflammation in asymptomatic joints but lacks specificity. In our patient, CT scan findings were consistent with spinal stenosis, while MRI showed similar results and mentioned new changes related to discitis. The gold standard for diagnosis is MSU crystals under the microscope in the joint fluid aspirate (Figure 3). The deposits of uric acid crystals seen in our patient during lumbar disc aspiration led to the diagnosis of gouty spondylodiscitis [2,7].

In general, there is no significant difference in the management of gout involving peripheries or the spinal cord. In the acute setting, one can use steroids and non-steroidal anti-inflammatory agents (NSAIDs) rather than using allopurinol because it can aggravate the disease. In patients with CKD, gastric ulcers, and NSAID allergy, colchicine can be used. For prevention of further attacks, maintenance medications such as allopurinol and febuxostat are recommended. The goal is to maintain uric acid levels less than 6 mg/dL. During an acute attack, usually, allopurinol is avoided as it exacerbates the symptoms. Surgical intervention is indicated in patients with neurological deficits due to axial gout who are not responding to therapy [8]. Fortunately, our patient improved without surgery.

## Conclusions

Gouty spondylodiscitis is a very rare condition and can mimic infectious etiologies. A back pain presentation with a previous history of gout should raise suspicion for axial involvement. A high index of suspicion and awareness is needed to diagnose spinal cord involvement due to gout, as its presentation can vary from simple back pain to spinal cord compression and paraplegia. Imaging modalities are not very sensitive to early joint changes, and MSU crystal identification in joint aspirate is needed for definitive diagnosis. Prompt diagnosis and treatment are necessary to avoid neurological complications. Further advancement should find better noninvasive diagnostic techniques.
